# Supplementary figures and images for: HIV-1 Subtype C Drug Resistance Mutations in Heavily Treated Patients Failing Integrase Strand Transfer Inhibitor-Based Regimens in Botswana
Source: Viruses. 2021 Mar 31;13(4):594. doi: 10.3390/v13040594 (PMC8066386; doi:10.3390/v13040594)

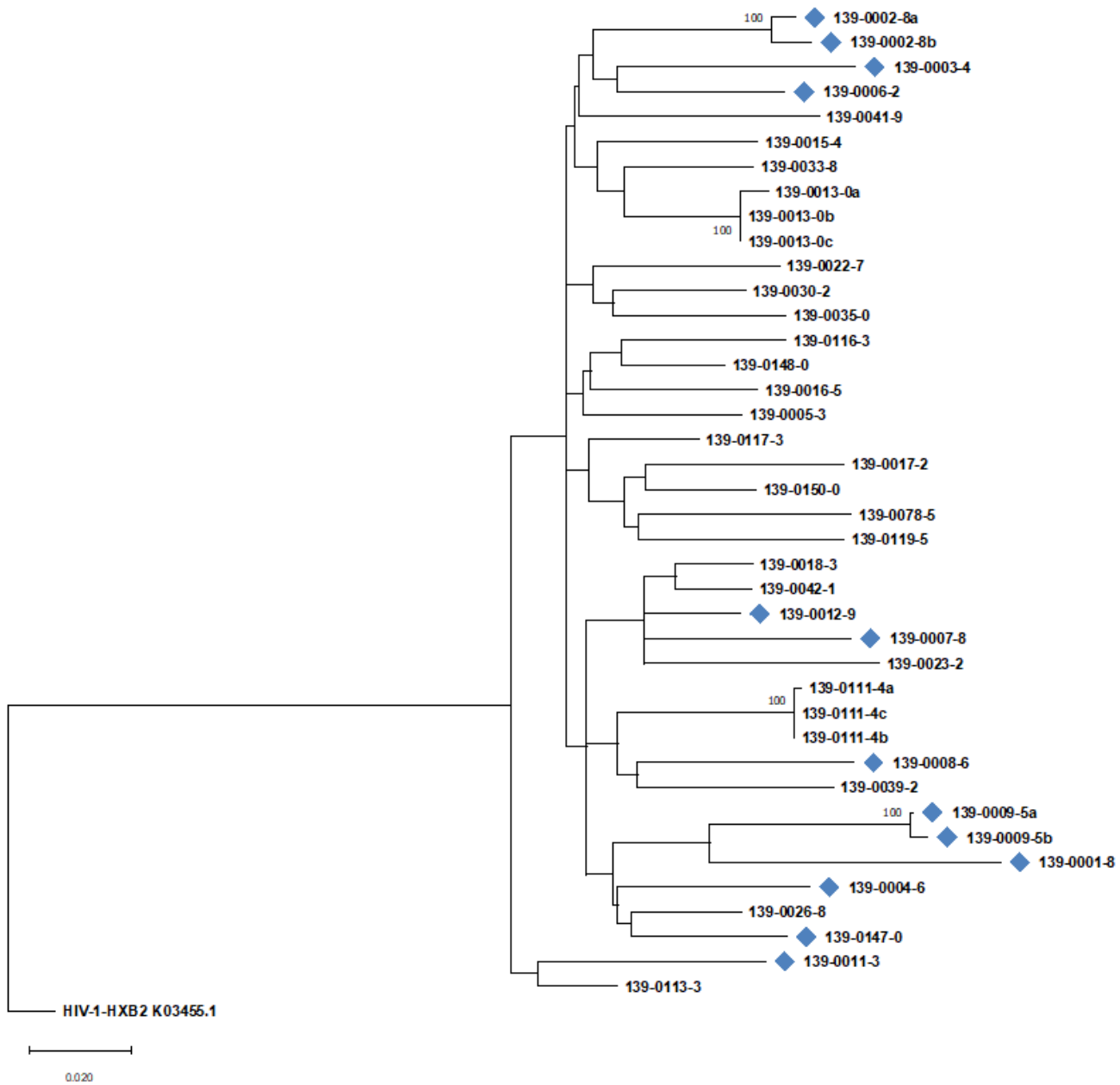

Supplement: Supplementary file 1 [file viruses-13-00594-s001.pdf]
